# Supplementary material for: How Does the Concentration of Determinants Affect Industrial Innovation Performance? – An Empirical Analysis of 23 Chinese Industrial Sectors
Source: PLoS One. 2017 Jan 18;12(1):e0169473. doi: 10.1371/journal.pone.0169473 (PMC5242457; doi:10.1371/journal.pone.0169473)
Supplement: S1 File — (DOCX) [file pone.0169473.s001.docx]

**How Does the Concentration of Determinants Affect Industrial Innovation Performance? – An Empirical Analysis of 23 Chinese Industrial Sectors**

*Shansong Huang* *^1 2^*, *Yang Bai* *^3^* ^[[1]](#footnote-2)^*, *Qingmei Tan* *^1^*

*^1^* *College of Economics and Management, Nanjing University of Aeronautics and Astronautics, 29 Yudao Street, 211106, Nanjing, China.*

*^2^* *Guangxi University of Science and Technology, 268 Donghuan Road,* *545006, Liuzhou, China.*

*^3^ School of Business, Nanjing Normal University,* *1 Wenyuan Road, 210023, Nanjing, China.*

# Abstract

The agglomeration of innovation determinants has a significant influence on the innovation performance of industries and enterprises. Such an effect has received less attention in empirical research studies. This study involves a survey of the agglomeration effect of two important innovation determinants, *R&D* investment and *R&D* personnel, and its influence on innovation performance from the perspective of the industrial level. We analysed the agglomeration features based on the panel data of 23 Chinese industrial sectors from 2001~2013. An interpretation model is proposed to examine the agglomeration effect on innovation performance for 4 industrial groups: state-owned enterprises, individual enterprises, foreign-owned enterprises and enterprises as a whole. We found two main results. First, the agglomeration of determinants has a clear positive effect on the innovation performance of all 4 groups but affects individual enterprises more significantly, followed by state-owned and foreign-owned enterprises. Second, the state-owned enterprises show a much higher concentration of *R&D* investment and *R&D* personnel than other groups. However, the induced innovation efficiency in the state-owned enterprises is worse than in the individual enterprises. The advantage of resources and capital does not translate into corresponding innovation output. The privately owned small and medium-sized enterprises (SMEs) show a high capability of technological innovation and mercerization but have limited innovation resources.

**Key words**: Innovation-determinant agglomeration; Innovation performance; *R&D* investment; *R&D* personnel; China industrial sectors

# 1. Introduction

The Chinese government is now making great efforts to maintain its economic growth in a so-called “New Normal” state, which refers to expectations of 6%~7% GDP growth rates in China for the foreseeable future [1]. As emphasized by Prime Minister Li, the key solution is to cultivate a new economic momentum and promote innovation. According to the Global Competitiveness Report 2015-2016 published by The World Economic Forum, the Global Competitiveness Index of China ranks 28th in the word and 9th in the Asian region. The innovation and sophistication factor, the innovation capacity and the technology readiness level^[[2]](#footnote-3)^, which reflect the competitiveness of technology and innovation directly, rank only as 34, 36 and 74, respectively, of all 138 countries. [3]. It is obvious that the relatively lower technology and innovation capacity has dragged down national competitiveness.

To promote innovation activities, the Chinese government has been increasing its financial investment and policy support during the last decades. According to the OECD Science Technology and Industry Outlook 2014, the gross domestic expenditure on *R&D* (GERD) increased more than 500% from $47,504.42 million (PPPs) in 2002 to $29,3549.52 million (PPPs) in 2012. The GERD share of GDP increased from 1.07% to 1.98% during the same time [2]. The question is, why does increasing innovation expenditure not improve innovation performance? In addition, what are the essential determinants, and how do they influence innovation performance? Many studies have tried to answer these questions. Several earlier studies have identified some key innovation determinants, i.e., *R&D* expenditure, human resource, enterprise or industry property and public policy. Scherer [4] establishes the earliest linear regression model to examine the impact of *R&D* investment on innovation performance based on a data set of Fortune Global 500 Company. Nelson et al. [5] performs a systematic study of the national systems of technical innovation where the authors emphasize the important role of *R&D* investment and human resources. Lundvall [6] mentions that the critical elements include firm organization, *R&D*, and public sector policy, as well as the institutional structure of the financial sector. The OECD [7] proposed more institutional factors, i.e., an education and training system, a macro-economic and regulatory context, and communication infrastructures. In some of the literature, Nonaka and Takeuchi [8] suggest that ability of knowledge management plays a key role in the capability of a company to create new knowledge. Hemmert [9] performs a more specific empirical analysis of the influence of institutional factors on the innovation performance of German and Japanese pharmaceutical and semiconductor business units. Bettencourt et al. [10] develop a model that explains the nonlinear response of technological innovation to various types of investment.

In recent decades, scholars have shown more interest in the effect of the technology spillover effect [11-14]. Berchicci [15] investigated how the configuration of internal and external *R&D* processes influences a firm’s innovative performance and found that low external *R&D* firms can benefit to a greater degree with external *R&D*. Guan and Yam [16] provide some evidence regarding the contribution of international technology spillovers to the innovation performance of China’s high-tech industries.

Governments often play an important role in the process of innovation. The government could support firm innovation by financial incentives, regulatory provisions, and relevant policies [16]. There is much literature on the impact of government policies on and support of innovation. Storey and Techer [17] perform a survey of the influence of public policy measures implemented in European Union countries on entrepreneurship and innovation of new technology-based firms during the 1980s and early 1990s. Autant-Bernard et al. [18] emphasize the importance of regional innovation policies based on empirical results concerning localized knowledge spillovers within the European regions. Xing and Zhang [19] carried out a theoretical analysis and effectiveness test of China's innovation policy. Intarakumnerd and Chaminade [20] analyse the innovation policy and paradigms of Thailand and suggest that the practice follows old innovation paradigms and hardly addresses profound systemic problems. Yu [21] carried out a systematic empirical analysis of the determinants of enterprise innovation in which the influence of both external factors (i.e., infrastructure, tax, subsidies level or financial development level) and internal factors (i.e., physical, system and spirits) have been examined. However, the author found that the improvement of external factors such as fiscal, finances and the labour force has limited effect on enterprise innovation performance.

Among these studies, *R&D* investment and *R&D* personnel appear to be more important among all factors, whereas the relevance of political, regional factors and social structure also contribute to innovation performance [22]. Though there have been abundant studies focusing on various factors of their impact on innovation performance, we find the literature limited in the following aspects. First, empirical results seem to have difficulty arriving at a consensus. Some argue that increasing innovation inputs is a critical factor for innovation [23-25]. For different industries, it does not seem necessarily that increasing input relates to better innovation performance [26, 27]. To explain this inconsistency, there are still theoretical gaps to be filled. Second, though a great number of innovation determinants have been identified, the agglomeration effect of innovation determinants and its impact on the industrial innovation performance have rarely been analysed quantitatively. The present study fills this gap by promoting an empirical study of 23 Chinese manufacturing industries. Departing from previous research, this study tries to examine the driving force behind China’s innovation performance from a perspective of determinants agglomeration. A concentration of innovation determinants has been considered as the main explanatory variable for industrial innovation performance. We pay more attention to the heterogeneity of industries, which may also explain the divergence of empirical conclusions.

In the following parts, we begin by illustrating the measurement of industrial innovation performance and determinant convergence in section 2. Section 3 presents the methodology. Section 4 shows the empirical results and presents some discussion. Section 5 concludes the study with some policy recommendations.

# 2. Index and Data

## 2.1 Industrial innovation performance

Industrial innovation is an important part of a country’s national innovation system, and its driving force can come from different sources: *R&D* investment, technology transfer, and spillover effects from other industrial firms [28]. Although innovation performance is somewhat difficult to quantify and measure, its overall characteristics do not preclude the measurement of key dimensions of processes and outputs [29]. Generally speaking, emerging indicators represent innovation in two aspects: innovation inputs and innovation outputs. In particular, *R&D* has been widely used to measure the efforts of a country in terms of innovation input. The indicator is widespread mainly because of the availability of data. However, its significant limitations have also been well documented [30]. Moreover, *R&D* investment underpins technological activities in other functional areas such as design and production [31, 32]. Product sales have been widely used to indicate innovation achievement as innovation output [33, 34]. Geroski [35] argues that the growth of product sales is the crux of innovation. Scherer [4] observed that innovations typically do not increase marginal profit but increase product sales. After all, the motivation of innovation for enterprises is to obtain sustainable competitiveness and maximize revenue. This suggests that sales growth is a particularly meaningful indicator of post-innovation performance. Therefore, in this study, we select new product sales to represent innovation performance.

## 2.2 Concentration of innovation determinants

Many studies from the innovation literature have been carried out to determine which factors enhance innovative performance. It has been widely recognized that Research and Development (*R&D*) activities and *R&D* personnel have an essential impact on innovation performance. However, at the industrial level, we find little empirical evidence as to whether the concentration of *R&D* investment and *R&D* personnel contribute to the divergence of the innovation performance of different sectors. One of our objectives is to measure the concentration of *R&D* activities and *R&D* personnel in different sectors of China and examine its effect on the innovation performance at different industrial levels. To do so, a location quotients technique has been employed to examine the concentration of *R&D* investment and *R&D* personnel.

## 2.3 Data

This analysis is based on three different datasets. First, the *China Statistical Yearbook* *on Science and Technology* provides the dataset of *R&D* investment and technology investment for all sectors [36]. Second, we use the *China Statistical Yearbook 2014* dataset [37], which provides information regarding the quantity of employment for all sectors. The third source is the *China Industry Statistical Yearbook* *2014*, which provides information for the sales data for all sectors [38]. These datasets allow us to calculate the concentration of *R&D* investment and *R&D* personnel and compare the characteristics of innovation determinants. For all datasets, we selected their time series from 2001 to 2013. Due to the change of statistical methods and dimensions, we find that the classification of industrial sectors changed in 2012. Therefore, to ensure the reliability of results, we selected 23 sectors of the manufacturing industry^[[3]](#footnote-4)^. The 23 sectors are referred to as H1 to H23 (Table 1).

**Table 1.** Classification and Codes for industrial sectors

| Code | Sector |
| --- | --- |
| H1 | Processing of Food from Agricultural Products; |
| H2 | Manufacture of Foods; |
| H3 | Manufacture of Beverages; |
| H4 | Manufacture of Textiles; |
| H5 | Manufacture of Textile Wearing Apparel, Footwear and Caps; |
| H6 | Manufacture of Leather, Fur, Feather and Its Products; |
| H7 | Processing of Timber, Manufacture of Wood, Bamboo, Rattan, Palm, Straw; |
| H8 | Manufacture of Furniture; |
| H9 | Manufacture of Paper and Paper Products; |
| H10 | Processing of Petroleum, Coking, Processing of Nuclear Fuel; |
| H11 | Manufacture of Chemical Raw Material and Chemical Products; |
| H12 | Manufacture of Medicines; |
| H13 | Manufacture of Chemical Fibres; |
| H14 | Manufacture of Non-metallic Mineral Products; |
| H15 | Manufacture and Processing of Ferrous Metals; |
| H16 | Manufacture and Processing of Non-ferrous Metals; |
| H17 | Manufacture of Metal Products; |
| H18 | Manufacture of General Purpose Machinery; |
| H19 | Manufacture of Special Purpose Machinery; |
| H20 | Manufacture of Transport Equipment; |
| H21 | Manufacture of Electrical Machinery and Equipment; |
| H22 | Manufacture of Communication, Computer, Other Electronic Equipment; |
| H23 | Manufacture of Measuring Instrument, Machinery for Cultural and Office Work |

*Source: Standardization Administration of the People's Republic of China* (*SAC*)*, Classification and Code Standard of National Economy Industry (GB/T 4754-2011)*

# 3. Method

## 3.1 Location Quotient (*LQ*) index

In this study, we use a Location Quotient index to examine the concentration of essential innovation determinants in various sectors. The concentration will then be used as an explanatory variable in the empirical study. The location quotient (*LQ*) is a valuable way of quantifying how concentrated a particular industry, cluster, occupation, or demographic group is in a region compared to the nation. It has been widely utilized in geographical analysis since the 1940s. The *LQ* indicator can be simply thought of as a ratio that compares a region to a larger reference region according to some characteristic or asset. Here we consider the concentration of *R&D* personnel and *R&D* investment for each sector.

The location quotient index of *R&D* personnel for sector *i* can be calculated by equation (1),

$RDP_{i}=\frac{q'_{i}/q_{i}}{Q'/Q}$ (1)

where $q'_{i}$ is the *R&D* personnel input of sector *i*, *q_i_* is the whole employee of sector *i*, $Q_{i}^{'}$ is the *R&D* personnel input of all sectors, *Q_i_* is the whole employee input of all sectors, and *RDP_i_* indicates the concentration of *R&D* personnel for sector *i*. Similarly, we use *RDM_i_* to indicate the concentration of *R&D* investment for sector *i* as equation (2),

$RDM_{i}=\frac{p'_{i}/p_{i}}{P'_{i}/P_{i}}$ (2)

where $p'_{i}$ is the quantity of *R&D* investment of sector *i*, *p_i_* is the whole technology input of sector *i*, $P_{i}^{'}$ is quantity of *R&D* investment of all sectors, and *P_i_* is the whole technology input of all sectors. According to the National Bureau of Statistics of China, the total technology input is the gross expenditure of personnel, finance, materials, information and all the other activities associated with science and technology. Technology activity includes R&D, R&D achievements industrialization and service-related science and technology [36]. Statistically, the technology input includes *R&D* investment. In this article, the two indicators are distinguished. The advantage of the indicator *RDM* is in being able to compare differences in the innovation input of different industries, while moderating the effect of industry size.

## 3.2. Interpretation model

The objective of the study is to examine how the concentration of innovation determinants, *R&D* personnel and *R&D* investment affect the innovation performance of different industrial sectors. For this purpose, we collected the necessary data and calculated the concentration index for each of the innovation determinants. Based on our hypotheses, the paper established an econometric model where the concentration of *R&D* personnel (*RDP_i_*) and *R&D* investment (*RDM_i_*) are considered as essential factors affecting innovation performance. To avoid the influence of other factors, i.e., industry size (*SIZE*), foreign technology spillover effect (*HTP*) and policy influence (*STF*), we introduce these factors in the model as control variables. Then, the regression model can be described as equation (3),

$INDP_{it}=\alpha_{i}+\beta_{1i}RDP_{it}+\beta_{2i}RDM_{it}+\beta_{3i}Con\mathrm{var}_{it}+\mu_{it}+\varepsilon_{it}$ (3)

where sector index *i* = 1,2,…,23, time series index *t* = 1, 2,…,13, indicating year 2001 to 2013, *α*, *β* and *μ* are the coefficients to be estimated, *ε* is the error, and *Convar* indicates the other factors that may have significant influence on innovation performance.

Generally speaking, the configuration process of production factors is different from that of innovation factors. The innovation factors work on production by a more intermediate process and are susceptible to external factors. Former empirical studies found that the industrial size, technology spillover effect, policy influence and regional factors have a notable impact on innovation performance. To be more precise, we extend the regression model by bringing in more control variables as equation (4).

(4)

We have noticed that previous literature usually uses per capita indicators to measure innovation performance. Sun & Du [28] believe that the per capita indicator describes innovation performance more comprehensively. It clearly shows the variation in innovation tendency. However, the per capita indicator has limitations in identifying the substantive differences in industries. Therefore, we have used the aggregate dataset of sale quantity as input. Moreover, since innovation performance is highly related to the nature of the enterprise, we have also examined the influence of innovation factors on state-owned enterprises, individual enterprises and foreign-owned enterprises by models (5) to (7), respectively.

(5)

(6)

(7)

Fiscal incentives can increase *R&D* investment and enhance innovation performance [39]. There are several ways in which the government could encourage innovation activities, such as directing finance support, conducting the *R&D* program, or providing a tax-based subsidy [40]. *R&D* programmes are more often applied in strategic emerging industries and aim to obtain essential technical breakthroughs in these industries. Therefore, they are not generally applicable. A tax-based subsidy seems to be the market-oriented policy, as it leaves the choice of how to conduct and pursue *R&D* activities to the private sector itself [41]. However, we cannot identify the subsidy that is directly responsible for *R&D* activities. Additionally, the data set for such a subsidy that goes to industrial sectors is unavailable. Therefore, we use direct financing support as a substitute.

There are gross industrial outputs, gross assets and gross employees, which can be used to indicate industrial size. Here we use a per capita gross product (gross industrial output divided by the number of enterprises) to present industrial size (*SIZE*).

Technology spillovers have been a major topic in technology innovation literature for many decades. Many empirical studies appear to support the presence of a technology spillover effect. There are distinct types of technology spillovers. The first is when domestic industrial firms learn from foreign-invested firms by observation or by establishing business relations with the latter. The second is through labour turnover, as domestic employees move from foreign to domestic firms [42]. As observed by many economists, Chinese enterprises have a long experience with technology import, learning and absorption prior to independent innovation. Therefore, the foreign technology spillover effect has an important impact on the innovation performance of Chinese enterprises. Since it is difficult to measure the spillover effect directly, we use the high-tech production import ratio (HTP) as a substitute. The regional difference is another impact factor that may influence industrial innovation performance. The agglomeration effect may vary for different regions. We use a dummy variable to present the regional difference. The eastern region is indicated by index 1, and the central and western regions are indicated by 0.

## 3.3 Model test

All the datasets or their logarithmic forms passed a unit root test under the significance level of 0.01, which means the data are suitable to be applied to regression analysis. There are three main panel data analysis approaches: independently pooled panels, a fixed effect model and a random effect model. We use the *F-test* and the *Hausman test* to identify the suitable model form for our analysis. The test results as shown below suggest that we chose a fixed effects model as our estimation method (Table 2).

**Table 2.** Estimations of the Pooled Regression Model, the Fixed Effects Model and the Random Effects Regression Model

|  | Pooled Regression Model | Fixed Effects Model | | Random Effects Regression Model |
| --- | --- | --- | --- | --- |
| *RDM* | 0.0873^＊^  (1.8971) | 0.6615^＊＊＊^  (5.222) | | -0.0444  (-0.9511) |
| *RDP* | 0.0637^＊＊^  (2.2069) | 0.4213^＊＊＊^  (2.8175) | | 0.0473^＊^  (1.9028) |
| *SIZE* | 0.3254^＊＊＊^  (5.2945) | 0.2839^＊＊＊^  (0.8292) | | 0.2866^＊＊＊^  (5.1605) |
| *STF* | 0.5411^＊＊＊^  (14.126) | 0.4703^＊＊＊^  (2.4589) | | 0.5286^＊＊＊^  (15.9961) |
| *HTP* | 0.2846^＊＊＊^  (5.3299) | 0.1232^＊＊^  (4.8352) | | 0.2090^＊＊＊^  (4.3969) |
| *Constant term* | 2.4929^＊＊＊^  (10.4006) | 3.7521^＊＊＊^  (13.5257) | | 3.0036^＊＊＊^  (13.407) |
| *Prob (F-statistic)* | 0.000000 | 0.000000 | | 0.000000 |
| *F-statistic* | F=7.435＞F0.05(22,271)=1.582 | | | |
| *Hausman test* | H= 69.834100; Prob. 0.0000 | | | |
| *R-squared* | 0.8159 | | 0.8852 | 0.7563 |
| *Adjusted R-squared* | 0.8128 | | 0.8738 | 0.7521 |
| *Observed value* | 299 | | 299 | 299 |

# 4. Results and discussion

In this section, we first analyse the development trend and agglomeration effect of innovation determinants given the statistical description of the dataset. Then, the empirical results of the study are provided and discussed based on the panel data of 23 Chinese industrial sectors from 2001 to 2013.

## 4.1 Concentration of innovation determinants

According to the indicators and datasets proposed in section 2, we examined the concentration of *R&D* investment and *R&D* personnel for 23 sectors. The results are shown in Tables 3 and 4.

Generally speaking, there are 7 sectors whose *R&D* investment concentration are higher than average. They are Manufacture of Measuring Instrument, Machinery for Cultural and Office Work (H22), Manufacture of Transport Equipment (H20), Manufacture of Electrical Machinery and Equipment (H21), Manufacture and Processing of Ferrous Metals (H15), Manufacture of Chemical Raw Material and Chemical Products (H11), Manufacture of General Purpose Machinery (H18), and Manufacture of Special Purpose Machinery (H19). Most of these 7 sectors belong to capital-intensive or technology-intensive industries. This implies that *R&D* investment gravitates to capital-intensive and technology-intensive industries. Sector H22, Manufacture of Communication, Computer, and Other Electronic Equipment, shows the highest *R&D* investment concentration. On the contrary, Manufacture of Furniture (H8) has the lowest *R&D* investment concentration.

**Table 3.** Concentration of *R&D* investment (*RDM*)

| ***Rank on mean value*** | ***Sector*** | ***Mean*** | ***Minimum*** | ***Maximum*** | ***Standard deviation*** |
| --- | --- | --- | --- | --- | --- |
| 1 | **H22** | 0.3089 | 0.2128 | 0.4876 | 0.0937 |
| 2 | **H20** | 0.2103 | 0.1801 | 0.2584 | 0.0249 |
| 3 | **H21** | 0.1567 | 0.1396 | 0.197 | 0.0151 |
| 4 | **H18** | 0.0952 | 0.0866 | 0.1081 | 0.0065 |
| 5 | **H11** | 0.1086 | 0.0919 | 0.1194 | 0.0081 |
| 6 | **H15** | 0.1393 | 0.1016 | 0.1854 | 0.0229 |
| 7 | **H19** | 0.0772 | 0.058 | 0.0994 | 0.0133 |
| 8 | **H12** | 0.0557 | 0.0437 | 0.081 | 0.0108 |
| 9 | **H4** | 0.035 | 0.0271 | 0.0453 | 0.006 |
| 10 | **H14** | 0.0315 | 0.0241 | 0.0379 | 0.0042 |
| 11 | **H16** | 0.0539 | 0.0338 | 0.1446 | 0.0279 |
| 12 | **H23** | 0.0234 | 0.0176 | 0.0296 | 0.0036 |
| 13 | **H17** | 0.0238 | 0.014 | 0.0394 | 0.0079 |
| 14 | **H1** | 0.0173 | 0.0091 | 0.0296 | 0.0064 |
| 15 | **H3** | 0.0186 | 0.0142 | 0.0232 | 0.0025 |
| 16 | **H10** | 0.0166 | 0.0133 | 0.0267 | 0.0034 |
| 17 | **H2** | 0.0136 | 0.0105 | 0.0174 | 0.0023 |
| 18 | **H9** | 0.0168 | 0.0135 | 0.0283 | 0.0046 |
| 19 | **H13** | 0.0155 | 0.0114 | 0.0208 | 0.0028 |
| 20 | **H5** | 0.0084 | 0.0043 | 0.0127 | 0.0026 |
| 21 | **H6** | 0.0036 | 0.0009 | 0.0058 | 0.0012 |
| 22 | **H7** | 0.0043 | 0.0021 | 0.0096 | 0.0018 |
| 23 | **H8** | 0.0021 | 0.0006 | 0.0038 | 0.001 |

Fig 1. shows the agglomeration tendency of *R&D investment* for the top 6 sectors and last 6 sectors based on the rank of the mean value given by Table 3. Though the *R&D investment* concentration of sector H22 remains the highest of all other sectors, its concentration decreases significantly through the period. The concentration of Manufacture of Transport Equipment (H20) decreases slightly. The other sectors stay relatively steady during the period.

**Fig 1.** Agglomeration of *R&D* investment (*RDM*) (2001-2013)

Compared to *R&D* investment, the *R&D* personnel concentration appears to undergo more fluctuation during the sample period. The top 8 sectors are Manufacture of Measuring Instrument, Machinery for Cultural and Office Work (H22), Manufacture of Transport Equipment (H20), Manufacture of Electrical Machinery and Equipment (H21), Manufacture of General Purpose Machinery (H18), Manufacture of Chemical Raw Material and Chemical Products (H11), Manufacture and Processing of Ferrous Metals (H15), and Manufacture of Special Purpose Machinery (H19). Their concentrations are higher than the average. As shown in Table 4, the top sectors of Manufacture and Processing of Ferrous Metals (H15), Manufacture of Transport Equipment (H20), Manufacture of Electrical Machinery and Equipment (H21), Manufacture of Communication, Computer, Other Electronic Equipment (H22) are almost the same as the *R&D* investment concentration, varying only in rank. The lowest concentration goes to sectors H5, H6, H7, H8 and H9, which are all labour-intensive industries. According to Fig 2, we find that the concentration of sectors H22, H21, H18 and H11 has increased since 2005, when the Chinese government decided to increase the number of *R&D* personnel by 580% from 1.2 million to 7 million during the following 5 years.

**Table 4.** Concentration of *R&D* personnel (*RDP*)

| ***Rank on mean value*** | ***Sector*** | ***Mean*** | ***Minimum*** | ***Maximum*** | ***Standard deviation*** |
| --- | --- | --- | --- | --- | --- |
| 1 | **H22** | 0.3089 | 0.2128 | 0.4876 | 0.0937 |
| 2 | **H20** | 0.2103 | 0.1801 | 0.2584 | 0.0249 |
| 3 | **H21** | 0.1567 | 0.1396 | 0.197 | 0.0151 |
| 4 | **H15** | 0.0957 | 0.0481 | 0.1362 | 0.0294 |
| 5 | **H11** | 0.1 | 0.0403 | 0.1259 | 0.0211 |
| 6 | **H18** | 0.1137 | 0.0465 | 0.1384 | 0.0232 |
| 7 | **H19** | 0.0772 | 0.058 | 0.0994 | 0.0133 |
| 8 | **H12** | 0.0585 | 0.019 | 0.0774 | 0.0158 |
| 9 | **H16** | 0.0395 | 0.0175 | 0.0556 | 0.0092 |
| 10 | **H4** | 0.0472 | 0.0247 | 0.0729 | 0.0136 |
| 11 | **H14** | 0.0413 | 0.0169 | 0.0516 | 0.009 |
| 12 | **H17** | 0.0283 | 0.0092 | 0.0504 | 0.0123 |
| 13 | **H23** | 0.0234 | 0.0176 | 0.0296 | 0.0036 |
| 14 | **H3** | 0.0176 | 0.0074 | 0.0234 | 0.0041 |
| 15 | **H1** | 0.0181 | 0.0064 | 0.0258 | 0.0048 |
| 16 | **H9** | 0.0142 | 0.0058 | 0.0172 | 0.003 |
| 17 | **H10** | 0.0168 | 0.0087 | 0.0287 | 0.0071 |
| 18 | **H13** | 0.0126 | 0.0051 | 0.0202 | 0.0037 |
| 19 | **H2** | 0.0146 | 0.0058 | 0.0185 | 0.0036 |
| 20 | **H5** | 0.011 | 0.0047 | 0.02 | 0.0045 |
| 21 | **H7** | 0.0037 | 0.002 | 0.0056 | 0.0015 |
| 22 | **H6** | 0.0061 | 0.0026 | 0.0087 | 0.0018 |
| 23 | **H8** | 0.003 | 0.0006 | 0.0058 | 0.0016 |

**Fig 2.** Agglomeration of *R&D* personnel (*RDP*) (2001-2013)

**4.2 Innovation performance for 23 sectors**

We use the ratio of new product sales to main operation income to represent industrial innovation performance. Statistics show that innovation performance in 9 sectors is higher than average; these are Manufacture of Textiles (H4), Manufacture of Medicines (H12), Manufacture and Processing of Ferrous Metals (H15), Manufacture and Processing of Non-ferrous Metals (H16), Manufacture of General Purpose Machinery (H18), Manufacture of Special Purpose Machinery (H19), Manufacture of Transport Equipment (H20), Manufacture of Electrical Machinery and Equipment (H21), and Manufacture of Communication, Computer, and Other Electronic Equipment (H22). Except for sector H4, all the others are capital-intensive or technology-intensive industries. Sector H22 shows the best innovation performance, which provides strong evidence of the rapid development of related Chinese industries over the last two decades. Processing of Timber, Manufacture of Wood, Bamboo, Rattan, Palm, and Straw (H7) shows the worst innovation performance compared with other sectors. Some of the sectors show a steady growth trend, i.e., Manufacture of Special Purpose Machinery (H19) and Manufacture of Electrical Machinery and Equipment (H21). The innovation performance of sector H23 improved the fastest of all 23 sectors (Table 5).

**Table 5 Industrial innovation performance**

| ***Sector*** | ***Mean*** | ***Minimum*** | ***Maximum*** | ***Standard Deviation*** | ***Rank on mean value*** |
| --- | --- | --- | --- | --- | --- |
| 1 | **H20** | 7.8541 | 7.0202 | 8.3029 | 0.4367 |
| 2 | **H22** | 7.6979 | 5.9539 | 8.3832 | 0.7829 |
| 3 | **H21** | 7.6669 | 7.1246 | 8.1418 | 0.3223 |
| 4 | **H15** | 7.4028 | 6.1367 | 7.9016 | 0.5364 |
| 5 | **H18** | 7.3048 | 6.4628 | 7.8615 | 0.4603 |
| 6 | **H19** | 7.3038 | 6.7372 | 7.7705 | 0.3305 |
| 7 | **H11** | 7.2636 | 6.3959 | 7.9608 | 0.502 |
| 8 | **H4** | 7.0275 | 6.3724 | 7.6076 | 0.4062 |
| 9 | **H16** | 6.9454 | 5.855 | 7.7153 | 0.6104 |
| 10 | **H12** | 6.9137 | 6.1621 | 7.557 | 0.4463 |
| 11 | **H10** | 6.8036 | 6.0988 | 7.4227 | 0.3589 |
| 12 | **H14** | 6.7668 | 5.9468 | 7.3822 | 0.4322 |
| 13 | **H17** | 6.7209 | 5.823 | 7.4349 | 0.5021 |
| 14 | **H13** | 6.6532 | 5.8327 | 7.1788 | 0.4188 |
| 15 | **H9** | 6.5972 | 6.0042 | 7.1406 | 0.3828 |
| 16 | **H23** | 6.5565 | 5.1944 | 7.1731 | 0.6549 |
| 17 | **H1** | 6.5258 | 5.6986 | 7.3267 | 0.6074 |
| 18 | **H3** | 6.5072 | 5.8829 | 7.0545 | 0.3959 |
| 19 | **H5** | 6.4629 | 5.788 | 7.1693 | 0.4546 |
| 20 | **H2** | 6.4107 | 5.5845 | 7.0401 | 0.4794 |
| 21 | **H6** | 6.2723 | 5.6052 | 6.8686 | 0.4082 |
| 22 | **H7** | 5.9057 | 4.8246 | 6.5258 | 0.5639 |
| 23 | **H8** | 5.8745 | 4.952 | 6.5923 | 0.5576 |

## 4.3 Influence of determinants concentration

This section examines the impact of the determinant concentration on industrial innovation performance, while taking into account the influence of financial support, industrial size, technology-absorptive capacity and regional difference. For this purpose, we take 23 industrial sectors’ time series data from 2001 to 2013 as input, then estimate and test the validation of the model and parameters. To identify the difference of the agglomeration effect on different industrial groups, we use four sample groups to represent state-owned enterprises, individual enterprises, foreign-owned enterprises and all enterprises.

Table 6 shows the estimation results of equations (4) ~ (7). As shown in the table, the coefficients of *R&D* personnel (*RDP*) and *R&D* investment (*RDM*) pass the significance test in all 4 groups. This suggests that the agglomeration of innovation determinants has a significant effect on innovation performance in all 4 groups. Specifically, it appears that agglomeration affects individual enterprise more significantly, compared with state-owned and foreign-owned enterprises.

The result implies at least two aspects. First, the agglomeration of innovation factors is conducive to improving the innovation performance of Chinese enterprises. The results show that the innovation effect of agglomeration for Chinese enterprises is better than for foreign-owned enterprise. Therefore, promoting the agglomeration of innovation factors may help Chinese enterprises catch up with advanced foreign enterprises. Second, we find that the agglomeration effect for state-owned enterprises is much larger than that for individual enterprises. This is because the state-owned enterprises normally have a larger scale and more capital reserve, which allows for external technology and resource import. They are also much easier to access and to finance compared with individual enterprises in China. However, the induced innovation efficiency of state-owned enterprises is worse than of individual enterprises. One reason is that Chinese state-owned enterprises may have resources and capital advantage but have less motivation to promote the technological mercerization, which largely depresses innovation efficiency. Another reason is that state-owned enterprises have had a high enthusiasm for importing and imitating new technologies over the last two decades, but they normally fail to realize independent innovation. As for individual enterprises, their innovation activities are market-oriented. To win the competitive advantage, they not only introduce and learn new technologies but also pay much more attention to technological innovation and application. Therefore, there is more motivation for individual enterprises to maximize the output of existing *R&D* investment or personnel resources.

We also perform a horizontal comparison of the effect of *R&D* personnel (*RDP*) with that of *R&D* investment (*RDM*). In all four cases, we find that the agglomeration effect of *R&D investment* (*RDM*) has played a more important role than *R&D* personnel (*RDP*). It is not necessary that a high concentration of *R&D* personnel be related to high innovation performance. For example, universities and research institutions are places where one finds the greatest agglomeration of *R&D* personnel. However, there is still large gap to taking full advantage of intellectual resources and translating them into innovation output. This suggests that to improve innovation performance and translate innovative technology into productivity and competitiveness, the government should try to encourage and accelerate technology transformation and industrialization.

**Table 6 Coefficient Estimates for the Fixed Effects Model**

| Variables | Explained Variable *INDP* | | | |
| --- | --- | --- | --- | --- |
| Explanatory Variable | *INDP* | *SOINDP* | *PEINDP* | *FFEINDP* |
| *RDM* | 0.6615^＊＊＊^  （5.222） | 0.5231^＊＊＊^（2.1571） | 0.6746^＊＊＊^（1.9896） | 0.2056^＊＊＊^  （1.5297） |
| *RDP* | 0.4213^＊＊＊^  （2.8175） | 0.3808^＊＊^（3.0271） | 0.4367^＊＊^（2.172） | 0.2020^＊＊＊^  （1.7867） |
| *SIEZ* | 0.2839^＊＊＊^（0.8292） | -0.0142^＊＊＊^（-4.6384） | 0. 3100^＊＊＊^（4.1102） | 0.2307^＊＊＊^（4.1231） |
| *HTP* | 0.1232^＊＊^（4.8352） | -0.0398^＊＊＊^（-15.307） | 0.0223^＊＊＊^（3.5746） | -0.0090^＊＊＊^（-1.8456） |
| *STF* | 0.4703^＊＊＊^（2.4589） | 0.6145^＊＊＊^（8.0508） | -0.0371^＊＊＊^（-8.5718） | -0.0291^＊＊＊^（-8.5750） |
| *D* | 1 | 0 | 0 | 0 |
| *Constant term* | 3.7521^＊＊＊^（13.5257） | 0.2157^＊＊＊^（16.8400） | 0.2230^＊＊＊^（7.2762） | 0.2803^＊＊＊^（11.637） |
| *F-statistic* | 77.4146 | 13.1311 | 7.0423 | 5.7195 |
| *Prob* | 0.00000 | 0.00000 | 0.00000 | 0.00000 |
| *R-squared* | 0.8852 | 0.5668 | 0.4123 | 0.3630 |
| *Adjusted R-squared* | 0.8738 | 0.5236 | 0.3538 | 0.2995 |
| *Observed value* | 299 | 299 | 299 | 299 |

# 5. Conclusion

This paper undertakes an empirical study of the effect of innovation determinants. Different from previous studies, i.e., Xing and Zhang [19], Intarakumnerd and Chaminade [20] and Yu [21], this study focuses on the agglomeration effect of innovation determinants. For this purpose, the following works have been implemented based on the panel data of 23 Chinese industrial sectors from 2001~2013. First, we calculated the agglomeration effect of two important innovation determinants, *R&D* investment and *R&D* personnel. It is found that technology-intensive and capital-intensive industries have shown a high agglomeration of innovation determinants over the past two decades, while the concentration of the labour-intensive industry has decreased significantly. Second, we established an empirical model, where the concentration of *R&D* personnel and *R&D* investment are considered as essential innovation determinants affecting innovation performance, while other factors, i.e., financial support, industrial size and technology spillover effects, are considered as well. The model has been used to estimate the impact of the determinant concentration on the innovation performance of 4 different industrial groups: state-owned enterprises, individual enterprises, foreign-owned enterprises and enterprises as a whole. Generally speaking, the impact of agglomeration is significant for all 4 groups, as shown by the results. However, it appears that agglomeration affects individual enterprises most significantly, followed by state-owned and foreign-owned enterprises. As far as different determinants are concerned, we find that the agglomeration of *R&D* investment has a more impulsive effect on industrial innovation than that of *R&D* personnel.

We learn the following implications from the statistics and empirical results. First, China’s industrial innovation efficiency has improved in the past two decades, but the capability of independent innovation still needs to be fostered, especially in state-owned enterprises. The financial support, industrial size and technology spillover effects still have a significant positive impact on innovation performance. The motivation of innovation determinant agglomeration has not been fully released. Second, with the advantage of resources and capital, Chinese state-owned enterprises show a much higher *R&D* personnel and *R&D* investment concentration. However, their induced innovation efficiency is worse than individual enterprises. This may be because in large enterprises, scale and market power have dampened motivation for innovation. On the other hand, privately owned SMEs show a high enthusiasm for technological innovation and mercerization, but fewer financial and personnel sources. In China, the SMEs play an important role in the economy. They account for more than 99% of all firms and contribute greatly to employment. To improve innovation performance across the board, SMEs still need to receive incentives and be supported. Third, the concentration of *R&D* personnel shows less of a contribution to innovation performance. To translate intellectual resources into productivity and competitiveness, we suggest that the government encourage the cooperation of enterprises and university and research institutions to accelerate technology transformation and industrialization.

# Acknowledgements

This work was supported by the National Natural Science Foundation of China under Grant 71403131; and Social Science Foundation of China under Grant No.15BGL029.

# Reference

1. Yang T, Long R, Li W, et al. (2016) Innovative Application of the Public–Private Partnership Model to the Electric Vehicle Charging Infrastructure in China. Sustainability 8: 738.
2. OECD (2016) Main Science and Technology Indicators Database. Paris.
3. The World Economic Forum (2016) The Global Competitiveness Report (2015-2016).
4. Scherer FM (1965) Firm size, market structure, opportunity, and the output of patented inventions. The American Economic Review 55: 1097-1125.
5. Nelson RR, Phelps ES (1966) Investment in humans, technological diffusion, and economic growth. The American Economic Review, 56: 69-75.
6. Lundvall BA (1992) National systems of innovation: Toward a theory of innovation and interactive learning. Vol. 1. Anthem Press.
7. OECD (1999) Managing National Innovation Systems. Paris.
8. Nonaka I, Takeuchi H (1995) The knowledge-creating company: How Japanese companies create the dynamics of innovation. Oxford university press.
9. Hemmert M (2004) The influence of institutional factors on the technology acquisition performance of high-tech firms: survey results from Germany and Japan. Research Policy 33: 1019-1039.
10. Bettencourt LMA, Trancik JE, Kaur J (2013) Determinants of the pace of global innovation in energy technologies. PloS one 10: e67864.
11. King DR, Dalton DR, Daily CM, et al. (2004) Meta‐analyses of post‐acquisition performance: Indications of unidentified moderators. Strategic management journal 25: 187-200.
12. Danzon PM, Epstein A, Nicholson S (2004) Mergers and acquisitions in the pharmaceutical and biotech industries. National Bureau of Economic Research.
13. Parida V, Westerberg M, Frishammar J (2012) Inbound open innovation activities in high‐tech SMEs: the impact on innovation performance. Journal of Small Business Management 50: 283-309.
14. West J, Bogers M (2014) Leveraging external sources of innovation: a review of research on open innovation. Journal of Product Innovation Management 31: 814-831.
15. Berchicci L (2013) Towards an open R&D system: Internal R&D investment, external knowledge acquisition and innovative performance. Research Policy 42: 117-127.
16. Guan JC, Yam RCM (2015) Effects of government financial incentives on firms’ innovation performance in China: Evidences from Beijing in the 1990s. Research Policy 44: 273-282.
17. Storey DJ, Tether BS (1998) Public policy measures to support new technology-based firms in the European Union. Research policy 9: 1037-1057.
18. Autant-Bernard C, Fadairo M, Massard N (2013) Knowledge diffusion and innovation policies within the European regions: Challenges based on recent empirical evidence. Research Policy 1: 196-210.
19. Xing F, Zhang JH (2009) Innovation policy in China: theoretical analysis and effectiveness test. Modern Economic Science 4: 63–69.
20. Intarakumnerd P, Chaminade C (2007) Strategy versus practice in innovation systems policy: the case of Thailand. Asian Journal of Technology Innovation 2: 197-213.
21. Yu ZP (2014) Determinants of technological innovation in China enterprises. Proceedings of the 11th international conference on innovation and management (ICIM 2014), Univ Vaasa, Vaasa, Finland, Vols I and II: 17-19.
22. Toole JL, Cha M, González MC (2012) Modeling the adoption of innovations in the presence of geographic and media influences. PloS one 1: e29528.
23. Falvey R, Foster N, Greenaway D (2004) Imports, exports, knowledge spillovers and growth . Economics Letters 85: 209-213.
24. Buckley PJ, Clegg J, Wang C (2007) Is the relationship between inward FDI and spillover effects linear? An empirical examination of the case of China. Journal of International Business Studies 38, 447-459.
25. Kafouros MI, Buckley PJ (2008) Under what conditions do firms benefit from the research efforts of other organizations? Research Policy 37: 225-239.
26. Haddad M, Harrison A (1993) Are there positive spillovers from direct foreign investment? Evidence from panel data for Morocco . Journal of development economics 42: 51-74.
27. Damijan JP, Knell M, Majcen B, et al. (2003) The role of FDI, R&D accumulation and trade in transferring technology to transition countries: evidence from firm panel data for eight transition countries. Economic systems 27: 189-204.
28. Sun Y, Du D (2010) Determinants of industrial innovation in China: Evidence from its recent economic census. Technovation 30: 540-550.
29. Fagerberg J (2005) The Oxford handbook of innovation. Oxford university press.
30. Patel P (2012) Indicators of innovation performance. In Tidd, J. (ed.) From Knowledge Management to Strategic Competence, London: Imperial College Press.

30. Bohn RE (1998) Measuring and managing technological knowledge. The Economic Impact of knowledge Butterworth-Heinemann, Boston 295-314.

31. Zhang J, Baden-Fuller C, Mangematin V (2007) Technological knowledge base, R&D organization structure and alliance formation: Evidence from the biopharmaceutical industry. Research policy 36: 515-528.

32. Stuart TE (2000) Interorganizational alliances and the performance of firms: A study of growth and innovation rates in a high-technology industry. Strategic management journal 21: 791-811.

33. Coad A, Rao R (2008) Innovation and firm growth in high-tech sectors: A quantile regression approach. Research policy 37: 633-648.

34. Geroski P (2002) The growth of firms in theory and in prattice. Competence governance and entrepreneurship 1: 168-186.

35. National Bureau of Statistics of the People's Republic of China (2015). China Statistical Yearbook on Science and Technology, Beijing.

36. National Bureau of Statistics of the People's Republic of China (2015). China Statistical Yearbook 2014, Beijing.

37. The national bureau of statistics of industrial statistics division (2015). China Industry Statistical Yearbook 2014, Beijing.

38. Hall B, Van RJ (2000) How effective are fiscal incentives for R&D? A review of the evidence. Research Policy 29: 449-469.

39. Klette TJ, Møen J, Griliches Z (2000) Do subsidies to commercial R&D reduce market failures? Microeconometric evaluation studies. Research Policy 29: 471-495.

40. Liu Z (2008) Foreign direct investment and technology spillovers: Theory and evidence. Journal of Development Economics 85: 176-193.

1. * *Corresponding author*

   *Email address*: *ybai@njnu.edu.cn*. [↑](#footnote-ref-2)
2. Technology readiness levels (TRL) are a method of estimating technology maturity of Critical Technology Elements (CTE) of a program during the acquisition process. [↑](#footnote-ref-3)
3. The statistics include only industrial enterprises with an annual revenue of 20 million RMB or more from their main business operations. [↑](#footnote-ref-4)
